# RETRACTION: Complications of Hidradenitis Suppurativa After Surgical Management: A Systematic Review and Meta‐Analysis

**DOI:** 10.1111/iwj.70595

**Published:** 2025-04-21

## Abstract

**RETRACTION:** TangB. , HuangZ. , YiQ. , and ZhengX. , “Complications of Hidradenitis Suppurativa After Surgical Management: A Systematic Review and Meta‐Analysis,” International Wound Journal 20, no. 4 (2023): 1253–1261, 10.1111/iwj.13945.36207796 PMC10031239

The above article, published online on 07 October 2022, in Wiley Online Library (http://onlinelibrary.wiley.com/), has been retracted by agreement between the journal Editor in Chief, Professor Keith Harding; and John Wiley & Sons Ltd. Following an investigation by the publisher, all parties have concluded that this article was accepted solely on the basis of a compromised peer review process. In addition, the investigation found textual overlap within the Introduction and Discussion sections between this article and another article by different authors [1]. The editors have therefore decided to retract the article. The authors did not respond to our notice regarding the retraction.

References

[1] MehdizadehA. , HazenP. G. , BecharaF. G. , et al., “Recurrence of Hidradenitis Suppurativa after Surgical Management: A Systematic Review and Meta‐Analysis,” Journal of the American Academy of Dermatology 73, no. 5, Supplement 1 (2015): S70–S77, 10.1016/j.jaad.2015.07.044.26470621

**RETRACTION:** B. Tang , Z. Huang , Q. Yi , and X. Zheng , “Complications of Hidradenitis Suppurativa After Surgical Management: A Systematic Review and Meta‐Analysis,” International Wound Journal 20, no. 4 (2023): 1253–1261, 10.1111/iwj.13945.36207796 PMC10031239

The above article, published online on 07 October 2022, in Wiley Online Library (http://onlinelibrary.wiley.com/), has been retracted by agreement between the journal Editor in Chief, Professor Keith Harding; and John Wiley & Sons Ltd. Following an investigation by the publisher, all parties have concluded that this article was accepted solely on the basis of a compromised peer review process. In addition, the investigation found textual overlap within the Introduction and Discussion sections between this article and another article by different authors [1]. The editors have therefore decided to retract the article. The authors did not respond to our notice regarding the retraction.

**References**

[1] A. Mehdizadeh , P. G. Hazen , F. G. Bechara , et al., “Recurrence of Hidradenitis Suppurativa after Surgical Management: A Systematic Review and Meta‐Analysis,” Journal of the American Academy of Dermatology 73, no. 5, Supplement 1 (2015): S70–S77, 10.1016/j.jaad.2015.07.044.26470621

